# Comments on “surface-based brain age index reflects hematological impacts on cognitive development in children with beta-thalassemia major”

**DOI:** 10.1016/j.isci.2026.117944

**Published:** 2026-10-26

Dear Editor,

We read with great interest the recently published article by Xu et al. titled “*Surface-based brain age index reflects hematological impacts on cognitive development in children with beta-thalassemia major*.”^1^ We sincerely appreciate the authors’ investigation into this critical clinical area, as well as their recognition of our previous work on the neurological impacts of beta-thalassemia. However, while we welcome the academic engagement, we are deeply concerned to notice that the findings attributed to our studies are the exact opposite of what our research reported and concluded.

In their introduction, Xu et al. state: “*This accumulation of iron adversely affects multiple organs, including the heart, liver, pancreas, and notably, the brain.*” To support this specific claim regarding brain iron accumulation, they directly cite our neuroimaging study.^2^ This assertion directly contradicts our published conclusions. In that paper, we explicitly stated: *“According to our MRI findings and the available histopathologic literature evidence, concerns about neural tissue iron overload in beta-thalassemia appear to be unjustified*.”^2^

Our findings are strongly supported by other refined MRI protocols and, most importantly, by the histopathologic study by Witzleben and Wyatt—currently the sole benchmark histopathologic evidence available in the literature—which reported absolutely no increased iron deposition in the neural tissue across all the five brains of adult beta-thalassemia patients examined, despite heavy systemic hemocromatosis.^3^ By citing our work to justify the exact opposite of our findings, the authors inadvertently mislead the readers and perpetuate a dangerous, reverberating myth in the literature. This misinformation stems from early, heterogeneous, and contradictory MRI observations that stand in open conflict with modern, robust MRI studies (such as Qiu et al.^4^) and histopathologic reality.

Regrettably, an identical pattern of miscitation occurs immediately after in the same paragraph. Xu et al. state that “*Recent findings have emphasized the impact of brain iron deposition on cognitive function in β-TM, leading to the recommendation that cognitive evaluations become a routine part of β-TM management*,” this time citing Tartaglione et al.^5^ Yet, Tartaglione et al. explicitly reported—even within the abstract—that “*no correlation was found with splenectomy, intracranial MRI/magnetic resonance-angiography findings, brain tissue iron content, or other disease-related clinical/laboratory/treatment data*.”

It is profoundly disappointing that two distinct papers concluding a lack of increased iron in neural tissue, or a lack of correlation with cognition performances, were used to claim the exact existence and cognitive impact of such accumulation.

Beyond miscitations, the manuscript suffers from some internal methodological flaws that merit to be mentioned.

1. Sample size issues: the overall sample size might be limited for the vast number of high-dimensional variables considered. The paper reports a surface-based brain age index (BAI) using graph neural networks to assess developmental delays in beta-thalassemia major. However, the relatively small sample size (*N* = 65–67) may limit the stability and generalizability of the analyses. In addition, the cross-sectional design does not allow causal mediation to be established between hematological factors and cognitive performance Furthermore, although validation in a large external sample of healthy individuals acquired using different MRI scanners provides some support for the generalizability of the model, it does not necessarily demonstrate adequate calibration in the target clinical population. Residual age-related regression bias, as well as differences in scanner characteristics, acquisition protocols, and population features, may influence BAI estimates. The interpretation of the observed brain-age difference as evidence of “delayed brain age” should therefore be made cautiously and supported by a clear assessment of prediction accuracy and calibration within the target dataset.
  To note, an internal inconsistency exists: the methods section claims the recruitment of 27 β-TM patients, yet the subsequent demographic tables and figures report data for only 25 patients. No drop-out report or exclusion criteria are provided anywhere to account for the disappearance of two subjects.
2. Inappropriate covariate corrections: the text mentions correcting for “postmenstrual age at scan” within this pediatric cohort (mean age about 9 years, range 6–15 years). For a study evaluating children, correcting for postmenstrual age is chronologically inappropriate for the vast majority of the sample (especially in beta-thalassemia pediatric subjects^6^ and for the male children included in the study).

Last, but not least, the authors utilize several advanced morphological metrics (such as cortical curvature and thickness) without reporting the actual directional findings. Is the curvature increased or decreased? Is the cortical thickness reduced or preserved? Instead, these biological metrics are compressed into a “predictability score” that entirely masks whether a specific metric is positively or negatively driving the BAI. We would suggest adding these basic data at least in the supplemental information helping hematologists and researchers to understand the true morphological changes occurring in the brain of beta-thalassemia patients. In fact, the complexity of the metrics reported makes the study difficult to interpret. Even Figure 5, reporting identical images both in healthy controls (first column) and patients (second column) for all the considered metrics seems to be poorly helpful, raising the question of how an un-interpretable map can serve as a clinical biomarker.

In summary, several inconsistencies make the paper difficult to translate into both research and clinical practice, especially for practicing physicians who require transparent, direction-specific biomarkers.

**Sincerely,**

**Renzo Manara**

**Alessandra Bertoldo**

**Silverio Perrotta**

**Immacolata Tartaglione**

https://doi.or/10.1016/j.isci.2026.117944

## Declaration of interests

The authors declare no conflict of interest.

## Declaration of generative AI and AI-assisted technologies in the manuscript preparation process

During the preparation of this work, the authors used ChatGPT for language checking. The authors reviewed and edited the output as needed and take full responsibility for the content of the published article.
